# Exploring arts-based interventions for youth substance use prevention: a scoping review of literature

**DOI:** 10.1186/s12889-022-14714-4

**Published:** 2022-12-06

**Authors:** Geoffrey Maina, Yiyan Li, Yiting Fang, Jonathan Amoyaw, Mamata Pandey, Thea Herzog, Daniel Nkrumah, Jordan Sherstobitoff, Ghazal Mousavian

**Affiliations:** 1 College of Nursing, University of Saskatchewan, Saskatoon, Canada; 2 University of Toronto, Toronto, Canada; 3 Dalhousie University, Halifax, Canada; 4 Saskatchewan Health Authority, Regina, Canada; 5 College of Arts and Science, University of Saskatchewan, Saskatoon, Canada; 6 College of Graduate and Postdoctoral Studies, University of Saskatchewan, Saskatoon, Canada

**Keywords:** Art-based intervention, Scoping review, Youth, Substance use

## Abstract

**Introduction:**

There is a rise in problematic substance use among Canadian youth, which is precipitating a public health crisis. Interventions are needed to empower youth to mitigate substance use risks. Active youth involvement in substance use prevention is urgently needed to increase uptake and ownership of the process and outcome of the intervention. Arts-based interventions are ideal participatory action approaches that can empower young people to be active agents in substance use prevention. These approaches can help promote health, reduce harm, and change behaviours. Scoping reviews are a vital tool that can help the research team identify relevant interventions that can be adapted to a community.

**Methods:**

This scoping review explores various arts-based substance use prevention interventions for youth. The scoping review used the iterative stages of Arksey and O’Malley to search Portal ERIC, Ovid MEDLINE, C.I.N.A.H.L., E.M.B.A.S.E., Web of Science, and A.P.A. PsycInfo and grey literature from Canadian Centre on Substance Use and Addiction and websites suggested by the Canadian Agency for Drugs and Technologies in Health. Inclusion criteria are a) articles utilizing arts-based intervention on substance use prevention; b) studies with a clearly defined intervention; c) intervention targeting the youth (age 12–17) and d) publications written in English. Thematic analysis was used to identify the main themes from the included articles.

**Results and discussion:**

Themes identified in a thematic synthesis of these studies included a) the intent of the intervention; b) intervention characteristics; and c) the perceived effectiveness of interventions. Art-based interventions increased knowledge and changed attitudes and practices on substance use among youth. Making the interventions aesthetically appealing and engaging, active youth involvement in the development of the intervention and developing youth-centred interventions which attended to the realities they faced were central to the success of these interventions.

**Supplementary Information:**

The online version contains supplementary material available at 10.1186/s12889-022-14714-4.

## Background

The use of illicit and licit substances such as alcohol, tobacco, cannabis, methamphetamines and opioids, continues to be a public health crisis in Canada [1]. Substance use has adverse effects on the quality of life of individuals and families while putting economic strain on society, costing over $46 billion, including $13.1 billion in health care costs [2, 3]. Substance use amongst youth in Canada is a growing problem. In 2017, nearly half of Canadian students in grades 7 to 12 (44%) reported consuming alcoholic beverages, and about 374,000 students in grades 7 to 12 (18%) reported using cannabis [4].

Prince Albert Saskatchewan has one of the highest youth substance use nationally, with a community report suggesting that about 73.8% of grade 10–12 students consume alcohol, which is 11% more than the national average [5]. Binge drinking among school-aged children in Prince Albert Region is estimated to be 67.9%, nearly 20% higher than the national average [6]. Moreover, children in the Prince Albert region are exposed to alcohol and drug use at an early age; from six to eleven years for alcohol, marijuana, opiates, and cocaine [7]. Consequences of this substance use are missing school, mental health, engagement with the criminal justice system and delinquency [7].

To address substance use among youth in this region, a consultative meeting comprising stakeholders in education, social and health sectors met in February 2018. This event led to a successful Collaborative Innovative Development grant from the Saskatchewan Health Research Foundation to actively engage affected youth in a photovoice project where school-going youth were to take photos to document their experiences and risks of substance use in their community. Due to public health measures occasioned by the COVID-19 pandemic, since photovoice is a form of an art-based intervention, undertaking a scoping review on art-based intervention would provide the research team with a diversity of interventions that could be implemented at a later date. Also, art-based interventions tend to actively engage youth in the research as change agents, a practice that has a positive impact on substance use prevention programs, thereby reducing substance use rates and overall morbidity and mortality [8].

Arts-based interventions use art as a medium to improve a process or a situation, especially one that involves emotional and psychological well-being [9]. Such interventions may include photography, poetic language, sculpture, painting, craft, music, and dance and can aid in the healing process and restoration [10]. Arts-based interventions focus on participants’ knowledge, experiences, and contributions by establishing an interactive environment and empowering dialogues between participants and their environments [11]. Arts-based interventions are widely used to promote health, reduce harm and change behaviours [12]. Art-Based interventions also provide an opportunity for participants to express their negative emotions and can help improve participants’ self-esteem and social inclusion [13, 14].

When used as substance use prevention interventions, art-based programs encourage youth participation, create an inclusive and safe environment and strengthen their power to face substance use issues [15]. Therefore, they can be empowerment tools that can help mitigate the risks of substance use thereby complementing other approaches for preventing and managing substance use disorders [16].Through art-based interventions, youth are empowered to resist substance use initiation by increasing knowledge, changing behaviours, and establishing self-confidence and self-esteem [17, 18].

This scoping review is intended to provide insight into how arts-based interventions for youth substance are designed and their perceived effectiveness. Information gathered from such a scoping review has the potential to provide insight to policymakers, program developers, and researchers with interventions surrounding substance use prevention for youth [19]. The findings of the scoping review will be presented to community stakeholders and will form the basis of the development of community-led culturally safe substance use prevention interventions for youth.

## Methods

A scoping review is undertaken to examine the extent, scope, and nature of research topics and identify gaps in the current literature [20]. This scoping review followed the steps identified by Arksey & O’Malley [21] and the results are reported using P.R.I.S.M.A. guidelines developed by Tricco et al., [22].

### Stage 1: identifying the research question

This review was based on three research questions: 1) What is known about arts-based interventions that prevent youth substance use; 2) What are the characteristics and study outcomes of these interventions, and 3) what are the perceived effectiveness of these interventions in preventing substance use among the youth?

### Stage 2: identifying relevant studies

Based on suggestions of a Health Sciences Librarian, researchers drafted the search strategy which centred on five keywords, including “arts-based”, “youth”, “substance use”, “intervention”, and “prevention.“ Databases, including ERIC, Ovid MEDLINE, C.I.N.A.H.L., E.M.B.A.S.E., Web of Science, and A.P.A. PsycInfo, were used to search for literature. Additionally, following the University of Toronto’s Grey literature searching guidelines, the team searched for grey literature from the websites suggested by the Canadian Agency for Drugs and Technologies in Health [23].

#### Inclusion criteria

The following four inclusion criteria were used to guide the screening of the articles to be included in the review: a) articles that used arts-based methods to prevent substance use among youth; b) studies that have a clearly defined intervention; c) studies that target youth 12- to 17-year-old; and d) written in English.

#### Exclusion criteria

Articles that were not written in English, whose focus was not on arts-based to prevent substance use, and those focussing outside of the age group 12 to 17 were excluded from the study. Also removed from the study are non-research publications such as reviews and letters to the editor.

### Stage 3- article selection

**Fig. 1:**
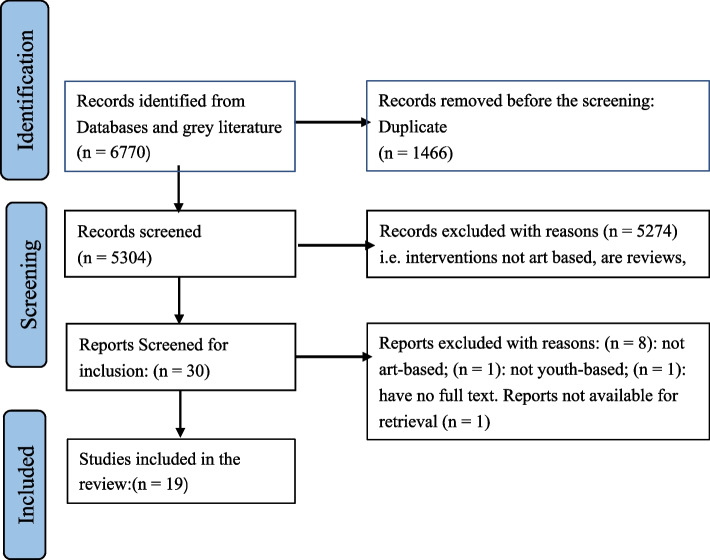
Prisma diagram

All articles sought from databases were input into Rayyan, an online platform that is used to screen and sort large numbers of references for inclusion in a review [24]. Two research team members- a postdoc and a research assistant identified and deleted duplicates and then independently screened articles for inclusion. A third person was involved to break a tie where there was no congruency regarding the inclusion or exclusion of an article or report. The screening process is summarised in the Prisma Diagram (Fig. 1).

A data extraction table was used to summarise the articles that were included in the scoping review using the following columns: article, study purpose, the intervention, Youth involvement, targeted level of intervention and implications for practice. Table 1 is a compilation of the studies that were included in the review.

**Table 1:** Compilation of the included articles

Article	Study Purpose	Arts-based intervention mode	Adjunct non-art-based intervention (lesson, curriculum, lecture)	Youth involvement and outcome
Bonyani, A., Safaeian, L., Chehrazi, M., Etedali, A., Zaghian, M., & Mashhadian, F. (2018). A high school-based education concerning drug abuse prevention. Journal of Education and Health Promotion, 7(1), 88.	To investigate the effectiveness of four educational methods on knowledge attitude and skills toward drug use.	Poster, leaflets Video	Lectures on life skills; training on drug abuse prevention; self-confidence skills; decision-making skills; cognitive skills; self-control skills; strategies for relieving stress and anxiety; social resistance skills.	**Youth involvement** Youth were involved in the pre-test, the pretest, and the evaluation of the programs. **The outcome of the intervention**: Change of attitudes toward drug abuse and addiction
Hecht, M. L., Corman, S. R., & Miller-Rassulo, M. (1993). An Evaluation of the Drug Resistance Project: A Comparison of Film Versus Live Performance Media. Health Communication, 5(2), 75–88.	To explore the effect of posttraining discussion on the effectiveness of film and live performance training media	Film/video and live performance	Performance modalities and one discussion agenda on drug resistance strategy; use of lived experiences, interviews and curriculum implementation on substance use prevention .	**Youth involvement** Youth validate the script and the approach used in the intervention. **The outcome of the intervention**: Change of attitude towards drug abuse; decreased perceived norms on drug use; belief in the ability to resist peer pressure
Polansky, J. M. (1994). Common and specific effects of substance-abuse-prevention videotapes on Mexican-American adolescents [Arizona]. https://www.proquest.com/docview/304122405?pq-origsite=gscholar&fromopenview=true	To provide the practitioner community with efficacy data, and the research community with information on whether any emerging treatment effects were consistent with the underlying intervention theory implicit in each video program.	Videotapes	A 10-item achievement test reflecting drug Knowledge was derived from the content of the information programming video. A similar 10-item Help Seeking questionnaire was constructed to reflect one’s disposition to select socially appropriate responses modelled in the help-seeking video.	**Youth involvement** Teens participated in experiments and finished all questionnaires. **The outcome of the intervention**: Increasing knowledge, helping skills and assertiveness to resist peer pressure Development/ reinforcement of conservative attitudes towards drugs and unwillingness to consume drugs
Warren, J. R., Hecht, M. L., Wagstaff, D. A., Elek, E., Ndiaye, K., Dustman, P., & Marsiglia, F. F. (2006). Communicating Prevention: The Effects of the keepin’ it REAL Classroom Videotapes and Televised PSAs on Middle-School Students’ Substance Use. Journal of Applied Communication Research, 34(2), 209–227. 10.1080/00909880600574153	To determine if exposure to two communication-oriented activities, videotapes and public service announcements, accounts for changes in substance use among adolescents participating in the Drug Resistance Strategies Project’s keepin’ it REAL adolescent substance use prevention curriculum.	Videotapes and televised series	Intervention emphasized REAL – Refuse, explain, avoid and leave. The curriculum comprised 10 lessons five of which entail forming video tapes. Other strategies used were billboards, televised PSAs and in-school booster sessions.	**Youth involvement** School teachers and teens helped project staff to develop a 10-lesson curriculum. With the help of teachers, students developed education videotapes. **The outcome of the intervention**: Participation in the study reduced the amount and frequency of marijuana and alcohol use
Stanley, L. R., Kelly, K. J., Swaim, R. C., & Jackman, D. (2018). Cultural Adaptation of the Be Under Your Own Influence Media Campaign for Middle-School American Indian Youth. Journal of Health Communication, 23(12), 1017–1025. 10.1080/10810730.2018.1536730	Using Be Under Your Own Influence (BUYOI) to prevent substance use among American Indian youth	Photovoice	15 students participated in a focus group to understand reservation life and BUYOI materials.	**Youth involvement** High school role models conducted photovoice. **The outcome of the intervention**: Reinforcing cultural identity and aspiration was protective against substance use
Duncan, T. E., Duncan, S. C., Beauchamp, N., Wells, J., & Ary, D. V. (2000). Development and evaluation of an interactive CD-ROM refusal skills program to prevent youth substance use: “refuse to use”. Journal of Behavioral Medicine, 23(1), 59–72. 10.1023/a:1005420304147	To provide materials that would extend and amplify the impact of traditional educational programs offered through the home, school, and other social organizations (e.g., health departments, religious organizations).	CD-ROM/Video	Focus groups and questionnaires	**Youth involvement** The youth was involved in providing information for the videotapes and providing feedback for the intervention. **The outcome of the intervention**: Personal efficacy to refuse an offer of marijuana Intention to refuse marijuana if offered Understanding social norms and respecting one’s decision to refuse a drug offer
Williams, C., Griffin, K. W., Macaulay, A. P., West, T. L., & Gronewold, E. (2005). Efficacy of a Drug Prevention CD-ROM Intervention for Adolescents. Substance Use & Misuse, 40(6), 869–878. 10.1081/JA-200042219	To examine the efficacy of substance abuse– preventive intervention using CD-ROM technology among adolescents in the sixth and seventh grade (12- to 13 years old).	CD-ROM	Students were required to complete the LST C D-ROM program within 6 weeks.	**Youth involvement** Students provided information for the pre-test and post-test, and parents signed the informed consent. **The outcome of the intervention**: Reduction in pro-drug attitudes Increased knowledge of relaxation skills, anxiety reduction skills, Increased knowledge of drugs
Hardoff, D., Stoffman, N., & Ziv, A. (2013). Empowering adolescents to control alcohol-associated risky situations. Archives of Disease in Childhood, 98(9), 672–675. 10.1136/archdischild-2013-303994	To describe and evaluate an experiential project which aims to augment existing alcohol high school educational programmes.	Lecture, enacted scenario and movie	A brief introductory lecture regarding alcoholic beverages, the immediate and late effects of alcohol, and legal regulations regarding blood alcohol levels and driving were provided. Two scenarios are enacted, one with a risky alcohol use scenario and the other one with the right response when in an environment where alcohol is used. A presentation of a movie regarding the consequences of alcohol use Emergency room scenario of a 16-year-old brought to the ER after a night of drinking	**Youth involvement** Youth attended a presentation by a person injured while driving under the influence of alcohol. **The outcome of the intervention**: Increased knowledge of the consequences of alcohol consumption Increased inclination to behavioural change
Shin, Y., Miller-Day, M., Hecht, M. L., & Krieger, J. L. (2018). Entertainment–Education Videos as a Persuasive Tool in the Substance Use Prevention Intervention “keepin’ it REAL”. Health Communication, 33(7), 896–906. 10.1080/10410236.2017.1321163	Evaluating the effects of entertainment education on adolescents’ alcohol prevention.	Video	Introductory video of REAL- Refuse, Explain, Avoid and Leave curriculum.	**Youth involvement** The video was produced and performed by high school students targeting middle school children. Aimed to teach resistance skills and change norms. **The outcome of the intervention**: Improved refusal self-efficacy which was inversely related to alcohol use behaviour Identification with the main character and resonating cultural context was associated with refusal of self-efficacy
Quek, L.-H., White, A., Low, C., Brown, J., Dalton, N., Dow, D., & Connor, J. P. (2012). Good choices, great future: An applied theatre prevention program to reduce alcohol-related risky behaviours during Schoolies. Drug and Alcohol Review, 31(7), 897–902. 10.1111/j.1465-3362.2012.00453.x	To investigate the effectiveness of Choices in reducing risky drinking, illicit drug use and problem behaviours (e.g. driving under the influence, arguments) during Schoolies.	Theatre/live acting	50 min theatre prevention program by CQCM students. Aimed to encourage students to make better choices and how they party and behave during the Schoolies. Safety messages include- alcohol and illicit drug use, seeking help, safe sex, drunk driving, liquor licence, policing services, and looking after mates. Safety messages embedded within skits, contemporary pop culture and music.	**Youth involvement** Students were given opportunities to interact and clarify issues raised by the program. **The outcome of the intervention**: The Choices program reduced the risk of illicit drug use
Turner-Musa, J. O., Rhodes, W. A., Harper, P. T. H., & Quinton, S. L. (2008). Hip-Hop to Prevent Substance Use and HIV among African American Youth: A Preliminary Investigation. Journal of Drug Education, 38(4), 351–365. 10.2190/DE.38.4.c	To examine the efficacy of a hip-hop based substance use and HIV prevention intervention that targets African- American middle-school youth.	Live action	An 88-item survey was used for the pre-test and post-test. was administered to all program participants.	**Youth involvement** Youth finished pre-test surveys and provided feedback via post-test. Parents signed the informed consent. **The outcome of the intervention**: Hip-hop can be an effective way to prevent risky behaviours, and participants have more perception of the dangers of substance use. A larger sample size could have yielded a significant difference between the study groups.
Duncan, L. R., Hieftje, K. D., Pendergrass, T. M., Sawyer, B. G., & Fiellin, L. E. (2018). Preliminary investigation of a videogame prototype for cigarette and marijuana prevention in adolescents. Substance Abuse, 39(3), 275–279. 10.1080/08897077.2018.1437862	To determine the preliminary efficacy of *smokeSCREEN* by exploring changes in knowledge, self-efficacy, attitudes, perceived norms, and intentions related to the use of cigarettes and marijuana as well as evaluate the overall experience of participants’ gameplay.	Videogame, named smokeSCREEN, contains avatars for players to choose from and decisions related to cigarettes and marijuana for them to make to get grades and social points. The video game involves cognitive and motivational variables to influence players’ thinking.	Participants played a smokeSCREEN, for one hour, twice a week. Pre and post-game assessment of knowledge, self-efficacy, attitudes, perceived norms, and intentions.	**Youth involvement** Participants finished pre-test surveys and post-test evaluations. **The outcome of the intervention**: The way knowledge about the effects of smoking and marijuana has an impact on the intention to use them. Video games turned out to be cognitively and motivationally impactful.
13–14 Duryea, E. J. (1983). Utilizing tenets of inoculation theory to develop and evaluate a preventive alcohol education intervention. The Journal of School Health, 53(4), 250–256. 10.1111/j.1746-1561.1983.tb01139.x Duryea, E., Mohr, P., Newman, I. M., Martin, G. L., & Egwaoje, E. (1984). Six-Month Follow-up Results of a Preventive Alcohol Education Intervention. Journal of Drug Education, 14(2), 97–104. 10.2190/5WR2-WTBY-C74F-LEFQ	1. To gain insight into questions surrounding inoculation-based Interventions and assess the extent to which the theory and specific variations of it are effective in meeting the objectives of a preventive alcohol education intervention. 2. To describe the long-term (6-month) effects of a high school (9th grade) alcohol education program designed to increase knowledge of alcohol’s effects upon performance, increase the ability of students to refute selected persuasive pro-drinking and driving arguments, and decrease the likelihood of complying with pressure to participate in risky alcohol-related situations.	Role-playing and slide show presentation	experiments, presentation. The Solomon Four-Group Design,	Youth participated in the project and provided feedback for initiative post-tests and six-month follow-up evaluations. **The outcome of the intervention** Preventive alcohol education programs are feasible and productive in schools. The project contained 4 parts: film, question and answer, role-playing exercises, and slide presentations. The movie provides information; the question and answer part allowed instructors to go through the main content of the movie; the role-playing enables students to be placed in alcohol-related situations and learn how to refuse the invitation of using substances from friends, adults, siblings and peers. During each role-playing, teachers also provided feedback to students. The presentations helped students renew and reinforce their knowledge.
van Leeuwen, L., Renes, R. J., & Leeuwis, C. (2013). Televised Entertainment-Education to Prevent Adolescent Alcohol Use. Health Education & Behavior, 40(2), 193–205. 10.1177/1090198112445906	To determine whether entertainment education (E-E) is a successful strategy for high school students and what processes may be involved.	TV	11 single-story televised episodes which are small movies with the storyline, characters, settings, themes and look and feel. 7 episodes were about alcohol, and the rest focus on cannabis, XTC, cocaine and multi-substance use.	Youth provided information for five online surveys, both pre-test and post-test. **The outcome of the intervention** Entertainment education (E-E) is an effective interventional strategy to decrease substance use behaviour among adolescents.
Huang, S. F., Zheng, W. L., Liao, J. Y., Huang, C. M., Lin, T. Y., & Guo, J. L. (2018). The effectiveness of a theory-based drama intervention in preventing illegal drug use among students aged 14–15 years in Taiwan. Health Education Journal, 77(4), 470–481. 10.1177/0017896918768647	To assess the effectiveness of a theory of planned behaviour (TPB)-based drama intervention for preventing drug use among young people aged 14–15 years.	Drama/Acting	Six 45 min sessions implemented over 6 weeks. Each session started with a warm-up activity, drama activity, conclusion and reflection. A conventional educational course was also included in the intervention- a 45-minute session on illegal drug prevention.	Students were involved in the projects and provided feedback for the intervention. **The outcome of the intervention** Change in subjective norms, attitudes, PBC and CI. Provided with life skills that helped them connect with the real world through role-play, and increased their self-efficacy in substance refusal skills
Safer, L. A., & Harding, C. G. (1993). Under pressure program: using live theatre to investigate adolescents’ attitudes and behaviour related to drug and alcohol abuse education and prevention. Adolescence, 28(109), 135–148. http://www.ncbi.nlm.nih.gov/pubmed/8456602	To describe the Under Pressure Program, an innovative communication-centred approach designed to involve Chicago public junior and senior high school students in considering the problems and prevention of adolescent substance abuse.	Theatre: a 30-minute musical play: Captain Clean	Participants were divided into the experimental group and the control group. Then, participants finished the pre-test and pro-test via a 20-item Likert scale about attitudes toward substance use.	Students participated in the whole project and provided information via discussion, pre-test and post-test. The Loyola University Center for Children and Families provided information for Parents and community stakeholders regarding the project, potential students’ needs and students’ reactions before the play and postperformance discussion among students. **The outcome of the intervention** Live theatre is a creative way for teachers to provide substance-using prevention information. Students watched the musical play, and then, participated in the postperformance group discussion by identifying characters and discussing characters’ points of view. Students felt empathy towards characters and realized the harms of substance use.
Harding, C. G., Safer, L. A., Kavanagh, J., Bania, R., Carty, H., Lisnov, L., & Wysockey, K. (1996). Using live theatre combined with role-playing and discussion to examine what at-risk adolescents think about substance abuse, its consequences, and prevention. Adolescence, 31(124), 783–796. http://www.ncbi.nlm.nih.gov/pubmed/8970653	Examine what at-risk adolescents think about substance abuse, consequences and prevention via live theatre.	Theatre: a 30-minute musical play: Captain Clean; role-playing	Quantitative ( questionnaire), qualitative (observation) and anecdotal methods.	Students attended a performance, and post-performance discussion, and finished post-test questionnaires. **The outcome of the intervention** After the intervention, students requested individual counselling to address drug abuse in their own lives or family The use of theatre, role play and discussion overcame barriers that inhibit effective communication between adults and youth. Drug refusal skills
de Visser, R. O., Graber, R., Hart, A., Abraham, C., Scanlon, T., Watten, P., & Memon, A. (2015). Using qualitative methods within a mixed-methods approach to developing and evaluating interventions to address harmful alcohol use among young people. Health Psychology, 34(4), 349–360. 10.1037/hea0000163	To illustrate how multiple qualitative methods can be combined and used within a resilience framework to develop and evaluate strengths-based behaviour change interventions to prevent harmful alcohol use among young people.	Sweet spot video	Survey; Interviews; focus group. A video-based resource was developed to promote responsible drinking among young people. Meant to illustrate a successful alcohol management strategy. Aim- to prompt individuals and groups to think critically about personal and social expectations and practices related to alcohol use	Youth participated in the project, providing feedback during 1 and 2, and that feedback influences the development of phases 2 and 3. Students provided information for phrase 4, the evaluation of the intervention. **The outcome of the intervention** Active engagement of young people, using peers as opinion leaders and models and encouraging young people to think critically about their behaviour.

### Stage 5- collating, summarizing, and reporting the results

The research team synthesized the collated findings by identifying common threads within the data. Rich narratives were developed to expound these threads that were thereafter designated as themes. P.R.I.S.M.A.‘s guidelines were used to report the screening process and its results. Thematic analysis, which entails familiarisation, coding, searching for themes, reviewing themes, defining and naming themes, and writing the report was applied [25].

### Stage 6- consulting

Presentations of the findings shall be made to community partners and stakeholders in the affected region after which an envisioning exercise shall ensue on how best to adapt the findings of the study to address substance use among the youth in the region.

## Results

Nineteen articles were included in this scoping review featuring diverse art-based interventions used to prevent substance use among youth.

Of these, nine used videos, six used theatre/acting modalities, a videogame and a photovoice project, respectively. Three themes were identified and are; a) the focus of the interventions; b) intervention vehicle characteristics; and c) why they were successful with the youth.

### Theme 1: The intent of the intervention

This theme focuses on what the intervention was intended to achieve to prevent substance use among youth. These interventions were designed to increase knowledge and change attitudes and practices on substance use. Changes in attitudes and practices were achieved through life skills training where youth were taught how to prevent substance use through the development of intrapersonal and interpersonal skills. Intrapersonal skills include improved decision-making skills, self-control, and strategies for dealing with anxiety or internal risks that would increase proclivities to substance use [26]. These skills increased the youth’s efficacy to manage risks associated with substance use [27] by fostering cultural identity and awareness [28]), and by imparting skills to make better choices that reduce substance use [29].

When participants developed interpersonal skills as a result of engaging with the intervention, they are empowered to challenge and resist potential danger posed by peers’ intent on introducing a substance to them. In so doing, they effectively dealt with substance use risks that confront them [30–32]. Refuse, explain, avoid, and leave (REAL) and assertiveness training was intended to reject substance use and peer pressure and to reinforce conservative attitudes towards substance use while reinforcing cultural identities [28, 33, 34] Subsequently, the youth were empowered to refuse invitations to use substances from friends, overcome peer pressure [35], and be prepared for real-world experiences [36].

Knowledge impartation was another focus of the interventions which entailed providing information on immediate and long-term impacts of substance use, regulations surrounding the use of alcohol and driving [37], and how to make better choices in risky environments [27, 29]. Other interventions provided advice on how to make socially acceptable responses when confronted with the offer of substances [34]. These interventions empowered the youth to critically think about the choices they made, and the impact of their decisions and endeavour to develop safe behaviours [37–39].

### Theme 2: Intervention characteristics

Intervention characteristics describe the mode of art-based interventions that were used in the substance use prevention project. In this review, we note that videos and live performances were the main modes that were used to deliver substance use prevention content to youth. Bonyani et al., [26] project used a video clip containing stories of people with lived experiences using substances. This approach increased awareness of commonly abused substances in Iran and the risk factors that predispose youth to substance use and the negative consequences associated with it. Hecht et al.‘s, [33] intervention utilized narrative accounts of personal resistance experiences which were categorized into the Refuse, Explain, Ask and Leave (REAL) strategy and thereafter adapted to a film where actors were coached to perform in a musical docudrama and the action was transferred to a film. The film was made in such a way that it appealed to teens.

Warren et al.‘s [40] project entailed creating a video by a local middle school performing arts group. The students conducted their interviews covering the four resistance strategies- Refuse, Explain, Ask and Leave (REAL) and thereafter took control of production, casting, music, dance, set, and postproduction. Duncan’s project [27] used information from focus group discussions with students to develop six vignettes to guide video production. Each vignette included a realistic and common situation relevant to refusal skills training for offers of marijuana. The refuse-to-use video was developed to get the youth to start thinking and communicating about drug use and peer pressure.

Williams et al., [41] project used a video intervention recorded on a CD-ROM modelled after Life Skills Training (L.S.T.) to teach general social skills, personal self-management skills, and resistance skills. It consisted of 10 sessions designed to be used in school and at home. The content was engaging using interactive audio and video content. Hardoff, Stoffman & Ziv [42] project used enacted scenarios performed by professional actors. They portrayed risky situations that a 15-year-old girl encounters with her 17-year boyfriend at a party. The boyfriend forces her to do things she does not want to do. Students then engage in a discussion regarding their feelings about the behaviours of the young couple. Quek et al.‘s, [29] applied a 50-minute theatre prevention program performed by volunteer students to encourage them to make better choices about how they party and behave during “schoolies”. The safety message embedded within skits, contemporary pop culture, and music was followed by a 20-minute discussion forum with student actors.

Turner-Musa et al.‘s, [32] intervention is a 10-lesson afterschool substance abuse and H.I.V. prevention live action using the contextual framework of youth popular culture. The intervention was implemented as a series of 10 two-hour sessions focusing on self-efficacy, norms, belief clarification, conflict resolution, and resistance. Duryea’s [35] project used role-playing exercises where students read prepared scripts which entailed enactment of situations where they were pressured to partake in risky behaviours. van Leeuwen et al.‘s, [37] project comprised 11 single-story episodes comprising of its storyline, characters, settings, theme and look and feel. Seven of these episodes focused on alcohol with the remainder focusing on other drugs. Huang et al.‘s, [36] intervention comprised six sessions of 45 min each, implemented over six weeks. It was made of warm-up activities, drama activities, a conclusion, and reflection. The drama activity entailed enactment, re-enactment, role-playing and acting the dialogue and scenario to integrate different components of life skills.

Safer & Harding, [43] project is a 32-minute theatre called Captain Clean comprised of the following content- general health concerns associated with drug and alcohol, dating relationships, peer/friend relationships, parent/family relationships, and counselling action. de Visser et al.‘s, [38] project entailed developing an 11-minute video called ‘hitting the sweet spot’ to promote responsible drinking among young people aimed at helping individuals critically think about personal and social expectations and practices related to substance use.

### Theme 3: Perceived effectiveness of interventions

In each project included in this review, the researcher evaluated the impact of the proposed intervention. In this theme, we report what these researchers reported as the perceived effectiveness of the implemented project. Many factors determined the perceived effectiveness of the interventions. These include-aesthetic appeal qualities, the ability to increase agency and active youth involvement. Presentations were deemed effective by the youth as the content and the presentations were found to be attractive to them and directly relevant and relatable. Most programs had a component that had a direct engagement with youth. For instance, in Hecht’s et al., [33] intervention, youth reflected, discussed, and provided input following the live performances. Polansky’s [34] intervention taught assertiveness skills which applied to them. Warren et al., [40] intervention comprised a structured curriculum which increased their knowledge. Stanley et al., [28] reinforced cultural identity and aspirations through photovoice. Duncan et al., [27] and Shin et al., [31] interventions sought to develop refusal skills, while Williams et al., [41] project sought to change attitudes towards drugs. Hardoff et al., [42] project empowered the youth through knowledge acquisition.

Active youth engagement was regarded as an important component for evaluating program effectiveness and took various forms. Youth were appealed to programs that allowed their voices to be heard through, implementation, discussions, and consultations were of great appeal to them [29, 35, 36] Stanley et al., [28] and Turner-Musa et al., [32]incorporated cultural elements into substance use prevention, which reinforced the identity of the participants. Duncan et al., [27] sought to appeal to their cognition and emotions, while van Leeuwen et al., [37] project used characters and settings that are relatable to youth. Other interventions were grounded in the lived experiences of people who were negatively impacted by substance use [33].

## Discussions

This scoping review aims at exploring arts-based interventions for preventing substance use among youth. Based on the results included in the review, it is evident that most intervention programs focus on changing perceptions, gaining refusal skills, and reducing potential harm brought by using substances. Behaviour change requires gaining knowledge, shifting attitudes, and the formation of behaviour patterns [27]. It is therefore imperative that youth be equipped with knowledge and skills to respond in social situations with increased risk for substance use [31].

To impact change the desired change in participants, the program appealed to their cognition, affect and behaviours, whose import can be appreciated by using the knowledge, attitudes, and practices (K.A.P.) theory, which can explain how individuals change behaviours. It posits that with knowledge acquisition, one has a greater inclination to change attitudes, which in turn may change behaviour [44]. Important knowledge includes not only that the harms associated with substances but also social scenarios and refusal skills when offered substances by peers [45]. To impart knowledge, the education materials must be interesting and engaging [36].

Active engagement of youth through a program that used videogames, movies, dramas and live performances with key messages on substance use was reported to be effective in capturing youth’s attention [26, 27, 32, 36, 40]. Moreover, active participation in the programs, such as through discussions, role-playing, reflections, and interviews can be effective in retaining youth interest. Knowledge can also be imparted to the youth through sharing of lived experiences on the impacts of substance use. Subsequently, youth can be empowered to engage and be in charge of their lives [46]. Empowering youth to express and create in ways that are meaningful to them enhances their cultural identity, which may also be protective against substance use [47]. Also, it helps young people to share their experiences, thoughts, and creativity [15].

The uniqueness of using art as a vehicle to deliver messages on substance use prevention is in its ability to make the intervention engaging, relaxing, fun, and helpful [48]. For the art interventions to be effective, the developer must consider the needs of the youth within their context and be relatable to their lives, culture, and experiences [32, 33]. Such a design makes it easy for the youth to be engaged and invested in the project. Developing youth-centred intervention programs ensure that they are modelled with common risks that youth face. Youth face pressures of tumultuous identity-seeking amidst constant media messaging, significant developmental changes, and ease of substance availability [49].

To ensure that an art-based program is relevant to the targeted youth, it is imperative to be cognizant of the other risks that they face. For instance, mental illnesses such as depression, anxiety, post-traumatic stress disorder, and bipolar are strongly associated with substance use [50]. Given that adolescence is a developmental phase where various mental health issues begin to present, this age is an optimal time to identify, diagnose, and manage mental illness or substance use concerns for the best outcomes [51]. A mental health support system must be in place as a form of substance use prevention measure. Actively engaging youth in intervention programs is critical to their success [34]. For example, in Be Under Your Own Influence strategy, Stanley et al., [28] observed that the active engagement of program beneficiaries is key to its success. This collaboration enhances programs’ effectiveness and builds the capacity to enhance agency to prevent substance use and lead to social change and development.

Throughout this scoping review, significant lessons can be learned about developing interventions for youth substance use prevention. First, impacting life skills training can have a positive impact on youth agencies to resist substance offers and thereby prevent substance use. Equipping youth with the skills and knowledge to make informed decisions about their actions is vital to prevent substance use. A preponderance of research has shown that prevention programs aimed at increasing social skills and providing positive support can be effective in reducing substance use among youth [52, 53]. Prevention programs that focus on social resistance skills training teach youth to identify social situations in which they are likely to be exposed to substance use, and how to avoid these high-risk situations. Such resistance training and problem-solving skills enable them to make good and informed decisions [45].

## Conclusion

Creative expressions such as art, videos, drama, and photovoice provide individuals with a medium to explore their thoughts, emotions, behaviours and actions [54]. Successful interventions must attend to the setting and characters of the community in which they seek to develop programs, including cultural identity, and the prevalent substances. Cultural sensitivity is an effective public health strategy which can greatly impact the outcomes of an intervention [55]. In addition, the intervention must be relatable to those it is meant to benefit [56–58]. This includes featuring characters and settings that are like the target community [59]. Youth need to be actively involved in any intervention concerning them. This involvement fosters ownership of the process and the outcome, hence increasing its efficacy [27, 40, 60]. The interventions must be intellectually and aesthetically stimulating. Programs directed at preventing youth substance use should be appealing and contemporary in nature [32, 61].

Effective youth substance use prevention interventions should use relevant language and audiovisual content familiar to them [15, 45]. Delivering prevention information through technological tools including CD-ROM and other electronic media can produce behavioural change among the youth [27, 41, 62] Care should be employed when adapting these programs as some studies have found some such as D.A.R.E. to be ineffective in substance use prevention when replicated in diverse settings [63, 64].

### Limitations of the review

This scoping review used rigorous and transparent methods throughout the entire process to retrieve several articles to answer the research questions. Multiple keywords were searched from selected electronic databases. The study had some limitations, notwithstanding these. As applies to most scoping reviews, this study did not assess the quality of the various studies. Since only articles published in English were included, potentially relevant articles may have been omitted from this study.

## Supplementary Information

**Additional file 1: Appendix 1.** Searching Strategies of the Database ERIC. **Appendix 2.** Searching Strategies of the Database Ovid MEDLINE. **Appendix 3.** Searching Strategies of the Database CINAHL (EBSCO). **Appendix 4.** Searching Strategies of the Database EMBASE. **Appendix 5.** Searching Strategies of the Database Web of Science. **Appendix 6.** Searching Strategies of the Database APA PsycInfo. **Appendix 7.** Searching Strategies of Grey Literature. 

## Data Availability

Not applicable.

## Abbreviations

SUD: Substance Use Disorder
C.C.S.A.: Canadian Centre on Substance Use and Addiction
C.A.D.T.H.: Canadian Agency for Drugs and Technologies in Health
REAL: Refuse, explain, avoid, and leave
L.S.T.: Life Skills Training
K.A.P.: knowledge, attitudes, and practices
P.T.S.D.: Post-traumatic stress disorder
