# Supplementary material for: Exploring arts-based interventions for youth substance use prevention: a scoping review of literature
Source: BMC Public Health. 2022 Dec 6;22:2281. doi: 10.1186/s12889-022-14714-4 (PMC9724351; doi:10.1186/s12889-022-14714-4)
Supplement: Supplementary file 1 — Additional file 1: Appendix 1. Searching Strategies of the Database ERIC. Appendix 2. Searching Strategies of the Database Ovid MEDLINE. Appendix 3. Searching Strategies of the Database CINAHL (EBSCO). Appendix 4. Searching Strategies of the Database EMBASE. Appendix 5. Searching Strategies of the Database Web of Science. Appendix 6. Searching Strategies of the Database APA PsycInfo. Appendix 7. Searching Strategies of Grey Literature. [file 12889_2022_14714_MOESM1_ESM.pdf]

APPENDIX 1: Searching Strategies of the Database ERIC
Database: ERIC <1965 to January 2021>
Search Strategy:

----

1 exp Art/ (5972)
2 (art* adj1 work*).mp. [mp=abstract, title, heading word, identifiers] (1034)
3 artist*.mp. [mp=abstract, title, heading word, identifiers] (9929)
4 artwork*.mp. [mp=abstract, title, heading word, identifiers] (1458)
5 photovoice*.mp. [mp=abstract, title, heading word, identifiers] (249)
6 photography.mp. [mp=abstract, title, heading word, identifiers] (5160)
7 (time* adj2 media).mp. [mp=abstract, title, heading word, identifiers] (136)
8 paint*.mp. [mp=abstract, title, heading word, identifiers] (4384)
9 sculpture*.mp. [mp=abstract, title, heading word, identifiers] (1070)
10 film*.mp. [mp=abstract, title, heading word, identifiers] (17635)
11 movie*.mp. [mp=abstract, title, heading word, identifiers] (2535)
12 video*.mp. [mp=abstract, title, heading word, identifiers] (40538)
13 danc*.mp. [mp=abstract, title, heading word, identifiers] (5226)
14 drama*.mp. [mp=abstract, title, heading word, identifiers] (18928)
15 music*.mp. [mp=abstract, title, heading word, identifiers] (24067)
16 theatre*.mp. [mp=abstract, title, heading word, identifiers] (2755)
17 theater*.mp. [mp=abstract, title, heading word, identifiers] (5831)
18 1 or 2 or 3 or 4 or 5 or 6 or 7 or 8 or 9 or 10 or 11 or 12 or 13 or 14 or 15 or 16 or 17 (117084)
19 exp Adolescent/ (0)
20 adolescen*.mp. [mp=abstract, title, heading word, identifiers] (74112)
21 teen*.mp. [mp=abstract, title, heading word, identifiers] (10616)
22 youth*.mp. [mp=abstract, title, heading word, identifiers] (66321)
23 19 or 20 or 21 or 22 (126464)
24 exp Substance-Related Disorders/ (0)
25 chemical dependence.mp. [mp=abstract, title, heading word, identifiers] (25)
26 drug abuse.mp. [mp=abstract, title, heading word, identifiers] (6902)
27 drug addiction.mp. [mp=abstract, title, heading word, identifiers] (1402)
28 drug dependence.mp. [mp=abstract, title, heading word, identifiers] (82)
29 "drug use disorder*".mp. [mp=abstract, title, heading word, identifiers] (16)
30 substance abuse*.mp. [mp=abstract, title, heading word, identifiers] (7675)
31 substance addict*.mp. [mp=abstract, title, heading word, identifiers] (20)
32 substance dependen*.mp. [mp=abstract, title, heading word, identifiers] (98)
33 "substance use".mp. [mp=abstract, title, heading word, identifiers] (2804)
34 cannabis.mp. [mp=abstract, title, heading word, identifiers] (243)
35 marijuana.mp. [mp=abstract, title, heading word, identifiers] (1866)
36 cocaine.mp. [mp=abstract, title, heading word, identifiers] (849)
37 cigarette*.mp. [mp=abstract, title, heading word, identifiers] (1487)
38 smok*.mp. [mp=abstract, title, heading word, identifiers] (5218)
39 alcohol.mp. [mp=abstract, title, heading word, identifiers] (10941)
40 "drug use".mp. [mp=abstract, title, heading word, identifiers] (5742)
41 meth*.mp. [mp=abstract, title, heading word, identifiers] (459491)

42 methamphetamine.mp. [mp=abstract, title, heading word, identifiers] (127)
43 Pharmaceutical misuse.mp. [mp=abstract, title, heading word, identifiers] (0)
44 24 or 25 or 26 or 27 or 28 or 29 or 30 or 31 or 32 or 33 or 34 or 35 or 36 or 37 or 38 or 39 or 40 or 41 or 42 or 43 (480152)
45 intervention.mp. [mp=abstract, title, heading word, identifiers] (78778)
46 prevent*.mp. [mp=abstract, title, heading word, identifiers] (45994)
47 preventive health services.mp. [mp=abstract, title, heading word, identifiers] (40)
48 [prevention control.fs.] (0)
49 46 or 47 or 48 (45994)
50 18 and 23 and 44 and 45 and 49 (65)
51 art therap*.mp. [mp=abstract, title, heading word, identifiers] (1026)
52 1 or 2 or 3 or 4 or 5 or 6 or 7 or 8 or 9 or 10 or 11 or 12 or 13 or 14 or 15 or 16 or 17 or 51 (117745)
53 23 and 44 and 45 and 49 and 52 (65)

****

APPENDIX 2: Searching Strategies of the Database Ovid MEDLINE

#	Search Statement	Results
1	exp art/	36049
2	(art* adj1 work*).mp. [mp=title, abstract, original title, name of substance word, subject heading word, floating sub-heading word, keyword heading word, organism supplementary concept word, protocol supplementary concept word, rare disease supplementary concept word, unique identifier, synonyms]	846
3	artist*.mp. [mp=title, abstract, original title, name of substance word, subject heading word, floating sub-heading word, keyword heading word, organism supplementary concept word, protocol supplementary concept word, rare disease supplementary concept word, unique identifier, synonyms]	7822
4	Artwork*.mp. [mp=title, abstract, original title, name of substance word, subject heading word, floating sub-heading word, keyword heading word, organism supplementary concept word, protocol supplementary concept word, rare disease supplementary concept word, unique identifier, synonyms]	1198
5	Art therap*.mp. [mp=title, abstract, original title, name of substance word, subject heading word, floating sub-heading word, keyword heading word, organism supplementary concept word, protocol supplementary concept word, rare disease supplementary concept word, unique identifier, synonyms]	2297
6	Arts-based----.mp. [mp=title, abstract, original title, name of substance word, subject heading word, floating sub-heading word, keyword heading word, organism supplementary concept word, protocol supplementary concept word, rare disease supplementary concept word, unique identifier, synonyms]	359
7	Photovoice*.mp. [mp=title, abstract, original title, name of substance word, subject heading word, floating sub-heading word, keyword heading word, organism supplementary concept word, protocol supplementary concept word, rare disease supplementary concept word, unique identifier, synonyms]	729
8	Photography.mp. [mp=title, abstract, original title, name of substance word, subject heading word, floating sub-heading word, keyword heading word, organism supplementary concept word, protocol supplementary concept word, rare disease supplementary concept word, unique identifier, synonyms]	37287
9	(Time* adj2 media).mp. [mp=title, abstract, original title, name of substance word, subject heading word, floating sub-heading word, keyword heading word, organism supplementary concept word, protocol supplementary concept word, rare disease supplementary concept word, unique identifier, synonyms]	721
10	Paint*.mp. [mp=title, abstract, original title, name of substance word, subject heading word, floating sub-heading word, keyword heading word, organism supplementary concept word, protocol	24255

	supplementary concept word, rare disease supplementary concept word, unique identifier, synonyms]	
11	Sculpture*.mp. [mp=title, abstract, original title, name of substance word, subject heading word, floating sub-heading word, keyword heading word, organism supplementary concept word, protocol supplementary concept word, rare disease supplementary concept word, unique identifier, synonyms]	2153
12	Film*.mp. [mp=title, abstract, original title, name of substance word, subject heading word, floating sub-heading word, keyword heading word, organism supplementary concept word, protocol supplementary concept word, rare disease supplementary concept word, unique identifier, synonyms]	183963
13	Movie*.mp. [mp=title, abstract, original title, name of substance word, subject heading word, floating sub-heading word, keyword heading word, organism supplementary concept word, protocol supplementary concept word, rare disease supplementary concept word, unique identifier, synonyms]	5815
14	Video*.mp. [mp=title, abstract, original title, name of substance word, subject heading word, floating sub-heading word, keyword heading word, organism supplementary concept word, protocol supplementary concept word, rare disease supplementary concept word, unique identifier, synonyms]	177546
15	Danc*.mp. [mp=title, abstract, original title, name of substance word, subject heading word, floating sub-heading word, keyword heading word, organism supplementary concept word, protocol supplementary concept word, rare disease supplementary concept word, unique identifier, synonyms]	8400
16	Drama*.mp. [mp=title, abstract, original title, name of substance word, subject heading word, floating sub-heading word, keyword heading word, organism supplementary concept word, protocol supplementary concept word, rare disease supplementary concept word, unique identifier, synonyms]	218843
17	Music*.mp. [mp=title, abstract, original title, name of substance word, subject heading word, floating sub-heading word, keyword heading word, organism supplementary concept word, protocol supplementary concept word, rare disease supplementary concept word, unique identifier, synonyms]	26944
18	Theatre*.mp. [mp=title, abstract, original title, name of substance word, subject heading word, floating sub-heading word, keyword heading word, organism supplementary concept word, protocol supplementary concept word, rare disease supplementary concept word, unique identifier, synonyms]	9274
19	Theater*.mp. [mp=title, abstract, original title, name of substance word, subject heading word, floating sub-heading word, keyword heading word, organism supplementary concept word, protocol supplementary concept word, rare disease supplementary concept	4005

	word, unique identifier, synonyms]	
20	1 or 2 or 3 or 4 or 5 or 6 or 7 or 8 or 9 or 10 or 11 or 12 or 13 or 14 or 15 or 16 or 17 or 18 or 19	711167
21	Teen*.mp. [mp=title, abstract, original title, name of substance word, subject heading word, floating sub-heading word, keyword heading word, organism supplementary concept word, protocol supplementary concept word, rare disease supplementary concept word, unique identifier, synonyms]	31213
22	Youth*.mp. [mp=title, abstract, original title, name of substance word, subject heading word, floating sub-heading word, keyword heading word, organism supplementary concept word, protocol supplementary concept word, rare disease supplementary concept word, unique identifier, synonyms]	83288
23	Adolescen*.mp. [mp=title, abstract, original title, name of substance word, subject heading word, floating sub-heading word, keyword heading word, organism supplementary concept word, protocol supplementary concept word, rare disease supplementary concept word, unique identifier, synonyms]	2148110
24	exp Adolescent/	2073753
25	21 or 22 or 23 or 24	2172863
26	exp Substance-Related Disorders/	284325
27	Chemical dependence*.mp. [mp=title, abstract, original title, name of substance word, subject heading word, floating sub-heading word, keyword heading word, organism supplementary concept word, protocol supplementary concept word, rare disease supplementary concept word, unique identifier, synonyms]	236
28	Drug abuse.mp. [mp=title, abstract, original title, name of substance word, subject heading word, floating sub-heading word, keyword heading word, organism supplementary concept word, protocol supplementary concept word, rare disease supplementary concept word, unique identifier, synonyms]	17838
29	Drug addiction.mp. [mp=title, abstract, original title, name of substance word, subject heading word, floating sub-heading word, keyword heading word, organism supplementary concept word, protocol supplementary concept word, rare disease supplementary concept word, unique identifier, synonyms]	8116
30	Drug dependence.mp. [mp=title, abstract, original title, name of substance word, subject heading word, floating sub-heading word, keyword heading word, organism supplementary concept word, protocol supplementary concept word, rare disease supplementary concept word, unique identifier, synonyms]	3980
31	"Drug use disorder*".mp. [mp=title, abstract, original title, name of substance word, subject heading word, floating sub-heading word, keyword heading word, organism supplementary concept word, protocol supplementary concept word, rare disease supplementary concept word, unique identifier, synonyms]	1187

32	Substance abuse*.mp. [mp=title, abstract, original title, name of substance word, subject heading word, floating sub-heading word, keyword heading word, organism supplementary concept word, protocol supplementary concept word, rare disease supplementary concept word, unique identifier, synonyms]	54123
33	Pharmaceutical misuse.mp. [mp=title, abstract, original title, name of substance word, subject heading word, floating sub-heading word, keyword heading word, organism supplementary concept word, protocol supplementary concept word, rare disease supplementary concept word, unique identifier, synonyms]	6
34	Substance addict*.mp. [mp=title, abstract, original title, name of substance word, subject heading word, floating sub-heading word, keyword heading word, organism supplementary concept word, protocol supplementary concept word, rare disease supplementary concept word, unique identifier, synonyms]	645
35	Substance dependen*.mp. [mp=title, abstract, original title, name of substance word, subject heading word, floating sub-heading word, keyword heading word, organism supplementary concept word, protocol supplementary concept word, rare disease supplementary concept word, unique identifier, synonyms]	2981
36	"Substance use".mp. [mp=title, abstract, original title, name of substance word, subject heading word, floating sub-heading word, keyword heading word, organism supplementary concept word, protocol supplementary concept word, rare disease supplementary concept word, unique identifier, synonyms]	37543
37	Cannabis.mp. [mp=title, abstract, original title, name of substance word, subject heading word, floating sub-heading word, keyword heading word, organism supplementary concept word, protocol supplementary concept word, rare disease supplementary concept word, unique identifier, synonyms]	23785
38	Marijuana.mp. [mp=title, abstract, original title, name of substance word, subject heading word, floating sub-heading word, keyword heading word, organism supplementary concept word, protocol supplementary concept word, rare disease supplementary concept word, unique identifier, synonyms]	20992
39	Cocaine.mp. [mp=title, abstract, original title, name of substance word, subject heading word, floating sub-heading word, keyword heading word, organism supplementary concept word, protocol supplementary concept word, rare disease supplementary concept word, unique identifier, synonyms]	43667
40	Cigarette*.mp. [mp=title, abstract, original title, name of substance word, subject heading word, floating sub-heading word, keyword heading word, organism supplementary concept word, protocol supplementary concept word, rare disease supplementary concept word, unique identifier, synonyms]	74158
41	Smok*.mp. [mp=title, abstract, original title, name of substance	336341

	word, subject heading word, floating sub-heading word, keyword heading word, organism supplementary concept word, protocol supplementary concept word, rare disease supplementary concept word, unique identifier, synonyms]	
42	Alcohol.mp. [mp=title, abstract, original title, name of substance word, subject heading word, floating sub-heading word, keyword heading word, organism supplementary concept word, protocol supplementary concept word, rare disease supplementary concept word, unique identifier, synonyms]	309303
43	"Drug use".mp. [mp=title, abstract, original title, name of substance word, subject heading word, floating sub-heading word, keyword heading word, organism supplementary concept word, protocol supplementary concept word, rare disease supplementary concept word, unique identifier, synonyms]	46360
44	Meth*.mp. [mp=title, abstract, original title, name of substance word, subject heading word, floating sub-heading word, keyword heading word, organism supplementary concept word, protocol supplementary concept word, rare disease supplementary concept word, unique identifier, synonyms]	10159486
45	Methamphetamine.mp. [mp=title, abstract, original title, name of substance word, subject heading word, floating sub-heading word, keyword heading word, organism supplementary concept word, protocol supplementary concept word, rare disease supplementary concept word, unique identifier, synonyms]	13749
46	26 or 27 or 28 or 29 or 30 or 31 or 32 or 33 or 34 or 35 or 36 or 37 or 38 or 39 or 40 or 41 or 42 or 43 or 44 or 45	10645880
47	Intervention.mp. [mp=title, abstract, original title, name of substance word, subject heading word, floating sub-heading word, keyword heading word, organism supplementary concept word, protocol supplementary concept word, rare disease supplementary concept word, unique identifier, synonyms]	644695
48	Prevent*.mp. [mp=title, abstract, original title, name of substance word, subject heading word, floating sub-heading word, keyword heading word, organism supplementary concept word, protocol supplementary concept word, rare disease supplementary concept word, unique identifier, synonyms]	2442591
49	Preventive.mp. [mp=title, abstract, original title, name of substance word, subject heading word, floating sub-heading word, keyword heading word, organism supplementary concept word, protocol supplementary concept word, rare disease supplementary concept word, unique identifier, synonyms]	157546
50	Preventive Health Services.mp. [mp=title, abstract, original title, name of substance word, subject heading word, floating sub-heading word, keyword heading word, organism supplementary concept word, protocol supplementary concept word, rare disease supplementary concept word, unique identifier, synonyms]	14319

51	prevention control.fs.	1325198
52	48 or 49 or 50 or 51	2442591
53	20 and 25 and 46 and 47 and 52	997

APPENDIX 3: Searching Strategies of the Database CINAHL (EBSCO)

#	Query	Limiters/Expanders	Last Run Via	Results
S53	S21 AND S26 AND S47 AND S48 AND S52	Expanders - Apply equivalent subjects Search modes - Boolean/Phrase	Interface - EBSCOhost Research Databases Search Screen - Advanced Search Database - CINAHL Plus with Full Text	2,052
S52	S48 OR S49 OR S50 OR S51	Expanders - Apply equivalent subjects Search modes - Boolean/Phrase	Interface - EBSCOhost Research Databases Search Screen - Advanced Search Database - CINAHL Plus with Full Text	1,195,526
S51	prevention control.fs.	Expanders - Apply equivalent subjects Search modes - Boolean/Phrase	Interface - EBSCOhost Research Databases Search Screen - Advanced Search Database - CINAHL Plus with Full Text	0
S50	preventive health services	Expanders - Apply equivalent subjects Search modes - Boolean/Phrase	Interface - EBSCOhost Research Databases Search Screen - Advanced Search Database - CINAHL Plus with Full Text	13,565
S49	prevent*	Expanders - Apply equivalent subjects Search modes - Boolean/Phrase	Interface - EBSCOhost Research Databases Search Screen - Advanced Search	845,849

			Database - CINAHL Plus with Full Text	
S48	intervention	Expanders - Apply equivalent subjects Search modes - Boolean/Phrase	Interface - EBSCOhost Research Databases Search Screen - Advanced Search Database - CINAHL Plus with Full Text	465,517
S47	S27 OR S28 OR S29 OR S30 OR S31 OR S32 OR S33 OR S34 OR S35 OR S36 OR S37 OR S38 OR S39 OR S40 OR S41 OR S42 OR S43 OR S44 OR S45 OR S46	Expanders - Apply equivalent subjects Search modes - Boolean/Phrase	Interface - EBSCOhost Research Databases Search Screen - Advanced Search Database - CINAHL Plus with Full Text	2,145,100
S46	Pharmaceutical misuse	Expanders - Apply equivalent subjects Search modes - Boolean/Phrase	Interface - EBSCOhost Research Databases Search Screen - Advanced Search Database - CINAHL Plus with Full Text	31
S45	methamphetamine	Expanders - Apply equivalent subjects Search modes - Boolean/Phrase	Interface - EBSCOhost Research Databases Search Screen - Advanced Search Database - CINAHL Plus with Full Text	3,960
S44	meth*	Expanders - Apply equivalent subjects Search modes - Boolean/Phrase	Interface - EBSCOhost Research Databases Search Screen - Advanced Search Database - CINAHL Plus with Full Text	1,912,625
S43	drug use	Expanders - Apply equivalent subjects Search modes - Boolean/Phrase	Interface - EBSCOhost Research Databases Search Screen - Advanced Search Database - CINAHL Plus with Full Text	58,678
S42	alcohol	Expanders - Apply equivalent subjects Search modes - Boolean/Phrase	Interface - EBSCOhost Research Databases Search Screen - Advanced Search	97,644

			Database - CINAHL Plus with Full Text	
S41	smok*	Expanders - Apply equivalent subjects Search modes - Boolean/Phrase	Interface - EBSCOhost Research Databases Search Screen - Advanced Search Database - CINAHL Plus with Full Text	125,056
S40	cigarette*	Expanders - Apply equivalent subjects Search modes - Boolean/Phrase	Interface - EBSCOhost Research Databases Search Screen - Advanced Search Database - CINAHL Plus with Full Text	24,935
S39	cocaine	Expanders - Apply equivalent subjects Search modes - Boolean/Phrase	Interface - EBSCOhost Research Databases Search Screen - Advanced Search Database - CINAHL Plus with Full Text	9,774
S38	marijuana	Expanders - Apply equivalent subjects Search modes - Boolean/Phrase	Interface - EBSCOhost Research Databases Search Screen - Advanced Search Database - CINAHL Plus with Full Text	12,364
S37	cannabis	Expanders - Apply equivalent subjects Search modes - Boolean/Phrase	Interface - EBSCOhost Research Databases Search Screen - Advanced Search Database - CINAHL Plus with Full Text	14,617
S36	substance use	Expanders - Apply equivalent subjects Search modes - Boolean/Phrase	Interface - EBSCOhost Research Databases Search Screen - Advanced Search Database - CINAHL Plus with Full Text	78,602
S35	substance dependen*	Expanders - Apply equivalent subjects Search modes - Boolean/Phrase	Interface - EBSCOhost Research Databases Search Screen - Advanced Search Database - CINAHL Plus with Full Text	12,105
S34	substance addict*	Expanders - Apply equivalent subjects	Interface - EBSCOhost Research Databases	1,457

		Search modes - Boolean/Phrase	Search Screen - Advanced Search Database - CINAHL Plus with Full Text	
S33	substance abuse*	Expanders - Apply equivalent subjects Search modes - Boolean/Phrase	Interface - EBSCOhost Research Databases Search Screen - Advanced Search Database - CINAHL Plus with Full Text	56,891
S32	drug use disorder	Expanders - Apply equivalent subjects Search modes - Boolean/Phrase	Interface - EBSCOhost Research Databases Search Screen - Advanced Search Database - CINAHL Plus with Full Text	31,364
S31	drug dependence	Expanders - Apply equivalent subjects Search modes - Boolean/Phrase	Interface - EBSCOhost Research Databases Search Screen - Advanced Search Database - CINAHL Plus with Full Text	9,138
S30	drug addiction	Expanders - Apply equivalent subjects Search modes - Boolean/Phrase	Interface - EBSCOhost Research Databases Search Screen - Advanced Search Database - CINAHL Plus with Full Text	9,152
S29	drug abuse	Expanders - Apply equivalent subjects Search modes - Boolean/Phrase	Interface - EBSCOhost Research Databases Search Screen - Advanced Search Database - CINAHL Plus with Full Text	30,183
S28	chemical dependency	Expanders - Apply equivalent subjects Search modes - Boolean/Phrase	Interface - EBSCOhost Research Databases Search Screen - Advanced Search Database - CINAHL Plus with Full Text	29,647
S27	(MH "Organic Mental Disorders, Substance-Induced+") OR (MH "Alcohol-Related	Expanders - Apply equivalent subjects Search modes - Boolean/Phrase	Interface - EBSCOhost Research Databases Search Screen - Advanced Search Database - CINAHL Plus with Full Text	173,120

	Disorders+") OR (MH "Substance Use Disorders+")			
S26	S22 OR S23 OR S24 OR S25	Expanders - Apply equivalent subjects Search modes - Boolean/Phrase	Interface - EBSCOhost Research Databases Search Screen - Advanced Search Database - CINAHL Plus with Full Text	598,456
S25	youth*	Expanders - Apply equivalent subjects Search modes - Boolean/Phrase	Interface - EBSCOhost Research Databases Search Screen - Advanced Search Database - CINAHL Plus with Full Text	54,143
S24	teen*	Expanders - Apply equivalent subjects Search modes - Boolean/Phrase	Interface - EBSCOhost Research Databases Search Screen - Advanced Search Database - CINAHL Plus with Full Text	21,229
S23	adolescen*	Expanders - Apply equivalent subjects Search modes - Boolean/Phrase	Interface - EBSCOhost Research Databases Search Screen - Advanced Search Database - CINAHL Plus with Full Text	581,830
S22	(MH "Adolescent Behavior") OR (MH "Adolescent Health")	Expanders - Apply equivalent subjects Search modes - Boolean/Phrase	Interface - EBSCOhost Research Databases Search Screen - Advanced Search Database - CINAHL Plus with Full Text	22,642
S21	S1 OR S2 OR S3 OR S4 OR S5 OR S6 OR S7 OR S8 OR S9 OR S10 OR S11 OR S12 OR S13 OR S14 OR S15 OR S16 OR S17 OR S18 OR S19 OR S20	Expanders - Apply equivalent subjects Search modes - Boolean/Phrase	Interface - EBSCOhost Research Databases Search Screen - Advanced Search Database - CINAHL Plus with Full Text	181,381
S20	art therap*	Expanders - Apply equivalent subjects Search modes - Boolean/Phrase	Interface - EBSCOhost Research Databases Search Screen - Advanced Search	8,991

			Database - CINAHL Plus with Full Text	
S19	theater*	Expanders - Apply equivalent subjects Search modes - Boolean/Phrase	Interface - EBSCOhost Research Databases Search Screen - Advanced Search Database - CINAHL Plus with Full Text	1,699
S18	theatre*	Expanders - Apply equivalent subjects Search modes - Boolean/Phrase	Interface - EBSCOhost Research Databases Search Screen - Advanced Search Database - CINAHL Plus with Full Text	4,642
S17	music*	Expanders - Apply equivalent subjects Search modes - Boolean/Phrase	Interface - EBSCOhost Research Databases Search Screen - Advanced Search Database - CINAHL Plus with Full Text	20,618
S16	drama*	Expanders - Apply equivalent subjects Search modes - Boolean/Phrase	Interface - EBSCOhost Research Databases Search Screen - Advanced Search Database - CINAHL Plus with Full Text	25,817
S15	danc*	Expanders - Apply equivalent subjects Search modes - Boolean/Phrase	Interface - EBSCOhost Research Databases Search Screen - Advanced Search Database - CINAHL Plus with Full Text	8,136
S14	video*	Expanders - Apply equivalent subjects Search modes - Boolean/Phrase	Interface - EBSCOhost Research Databases Search Screen - Advanced Search Database - CINAHL Plus with Full Text	68,717
S13	movie*	Expanders - Apply equivalent subjects Search modes - Boolean/Phrase	Interface - EBSCOhost Research Databases Search Screen - Advanced Search Database - CINAHL Plus with Full Text	4,614
S12	film*	Expanders - Apply equivalent subjects	Interface - EBSCOhost Research Databases	13,334

		Search modes - Boolean/Phrase	Search Screen - Advanced Search Database - CINAHL Plus with Full Text	
S11	sculpture*	Expanders - Apply equivalent subjects Search modes - Boolean/Phrase	Interface - EBSCOhost Research Databases Search Screen - Advanced Search Database - CINAHL Plus with Full Text	226
S10	paint*	Expanders - Apply equivalent subjects Search modes - Boolean/Phrase	Interface - EBSCOhost Research Databases Search Screen - Advanced Search Database - CINAHL Plus with Full Text	4,420
S9	time* adj2 media	Expanders - Apply equivalent subjects Search modes - SmartText Searching	Interface - EBSCOhost Research Databases Search Screen - Advanced Search Database - CINAHL Plus with Full Text	176
S8	time* adj2 media	Expanders - Apply equivalent subjects Search modes - Boolean/Phrase	Interface - EBSCOhost Research Databases Search Screen - Advanced Search Database - CINAHL Plus with Full Text	0
S7	photography	Expanders - Apply equivalent subjects Search modes - Boolean/Phrase	Interface - EBSCOhost Research Databases Search Screen - Advanced Search Database - CINAHL Plus with Full Text	12,687
S6	photovoice*	Expanders - Apply equivalent subjects Search modes - Boolean/Phrase	Interface - EBSCOhost Research Databases Search Screen - Advanced Search Database - CINAHL Plus with Full Text	774
S5	artwork*	Expanders - Apply equivalent subjects Search modes - Boolean/Phrase	Interface - EBSCOhost Research Databases Search Screen - Advanced Search Database - CINAHL Plus with Full Text	5,256

S4	artist*	Expanders - Apply equivalent subjects Search modes - Boolean/Phrase	Interface - EBSCOhost Research Databases Search Screen - Advanced Search Database - CINAHL Plus with Full Text	8,561
S3	art* adj1 work*	Expanders - Apply equivalent subjects Search modes - SmartText Searching	Interface - EBSCOhost Research Databases Search Screen - Advanced Search Database - CINAHL Plus with Full Text	55
S2	art* adj1 work*	Expanders - Apply equivalent subjects Search modes - Boolean/Phrase	Interface - EBSCOhost Research Databases Search Screen - Advanced Search Database - CINAHL Plus with Full Text	0
S1	(MH "Art+")	Expanders - Apply equivalent subjects Search modes - Boolean/Phrase	Interface - EBSCOhost Research Databases Search Screen - Advanced Search Database - CINAHL Plus with Full Text	16,472

APPENDIX 4: Searching Strategies of the Database EMBASE
Database: Embase Classic+Embase <1947 to 2021 April 02>
Search Strategy:
----
1 exp Art/ (73315)
2 (art* adj1 work*).mp. [mp=title, abstract, heading word, drug trade name, original title, device manufacturer, drug manufacturer, device trade name, keyword, floating subheading word, candidate term word] (1344)
3 artist*.mp. [mp=title, abstract, heading word, drug trade name, original title, device manufacturer, drug manufacturer, device trade name, keyword, floating subheading word, candidate term word] (9207)
4 artwork*.mp. [mp=title, abstract, heading word, drug trade name, original title, device manufacturer, drug manufacturer, device trade name, keyword, floating subheading word, candidate term word] (1430)
5 photovoice*.mp. [mp=title, abstract, heading word, drug trade name, original title, device manufacturer, drug manufacturer, device trade name, keyword, floating subheading word, candidate term word] (921)
6 photography.mp. [mp=title, abstract, heading word, drug trade name, original title, device manufacturer, drug manufacturer, device trade name, keyword, floating subheading word, candidate term word] (64307)
7 (time* adj2 media).mp. [mp=title, abstract, heading word, drug trade name, original title, device manufacturer, drug manufacturer, device trade name, keyword, floating subheading word, candidate term word] (1017)
8 paint*.mp. [mp=title, abstract, heading word, drug trade name, original title, device manufacturer, drug manufacturer, device trade name, keyword, floating subheading word, candidate term word] (33435)
9 sculpture*.mp. [mp=title, abstract, heading word, drug trade name, original title, device manufacturer, drug manufacturer, device trade name, keyword, floating subheading word, candidate term word] (1526)
10 film*.mp. [mp=title, abstract, heading word, drug trade name, original title, device manufacturer, drug manufacturer, device trade name, keyword, floating subheading word, candidate term word] (222910)
11 movie*.mp. [mp=title, abstract, heading word, drug trade name, original title, device manufacturer, drug manufacturer, device trade name, keyword, floating subheading word, candidate term word] (8832)
12 video*.mp. [mp=title, abstract, heading word, drug trade name, original title, device manufacturer, drug manufacturer, device trade name, keyword, floating subheading word, candidate term word] (221816)
13 danc*.mp. [mp=title, abstract, heading word, drug trade name, original title, device manufacturer, drug manufacturer, device trade name, keyword, floating subheading word, candidate term word] (11911)
14 drama*.mp. [mp=title, abstract, heading word, drug trade name, original title, device manufacturer, drug manufacturer, device trade name, keyword, floating subheading word, candidate term word] (285421)

15 music*.mp. [mp=title, abstract, heading word, drug trade name, original title, device manufacturer, drug manufacturer, device trade name, keyword, floating subheading word, candidate term word] (35871)
16 theatre*.mp. [mp=title, abstract, heading word, drug trade name, original title, device manufacturer, drug manufacturer, device trade name, keyword, floating subheading word, candidate term word] (18606)
17 theater*.mp. [mp=title, abstract, heading word, drug trade name, original title, device manufacturer, drug manufacturer, device trade name, keyword, floating subheading word, candidate term word] (5453)
18 1 or 2 or 3 or 4 or 5 or 6 or 7 or 8 or 9 or 10 or 11 or 12 or 13 or 14 or 15 or 16 or 17 (952075)
19 exp Adolescent/ (1729024)
20 adolescen*.mp. [mp=title, abstract, heading word, drug trade name, original title, device manufacturer, drug manufacturer, device trade name, keyword, floating subheading word, candidate term word] (1837476)
21 teen*.mp. [mp=title, abstract, heading word, drug trade name, original title, device manufacturer, drug manufacturer, device trade name, keyword, floating subheading word, candidate term word] (45373)
22 youth*.mp. [mp=title, abstract, heading word, drug trade name, original title, device manufacturer, drug manufacturer, device trade name, keyword, floating subheading word, candidate term word] (105204)
23 19 or 20 or 21 or 22 (1880307)
24 exp Substance-Related Disorders/ (263391)
25 chemical dependence.mp. [mp=title, abstract, heading word, drug trade name, original title, device manufacturer, drug manufacturer, device trade name, keyword, floating subheading word, candidate term word] (299)
26 drug abuse.mp. [mp=title, abstract, heading word, drug trade name, original title, device manufacturer, drug manufacturer, device trade name, keyword, floating subheading word, candidate term word] (81160)
27 drug addiction.mp. [mp=title, abstract, heading word, drug trade name, original title, device manufacturer, drug manufacturer, device trade name, keyword, floating subheading word, candidate term word] (11293)
28 drug dependence.mp. [mp=title, abstract, heading word, drug trade name, original title, device manufacturer, drug manufacturer, device trade name, keyword, floating subheading word, candidate term word] (70666)
29 "drug use disorder*".mp. [mp=title, abstract, heading word, drug trade name, original title, device manufacturer, drug manufacturer, device trade name, keyword, floating subheading word, candidate term word] (1569)
30 substance abuse*.mp. [mp=title, abstract, heading word, drug trade name, original title, device manufacturer, drug manufacturer, device trade name, keyword, floating subheading word, candidate term word] (71928)
31 substance addict*.mp. [mp=title, abstract, heading word, drug trade name, original title, device manufacturer, drug manufacturer, device trade name, keyword, floating subheading word, candidate term word] (896)

32 substance dependen*.mp. [mp=title, abstract, heading word, drug trade name, original title, device manufacturer, drug manufacturer, device trade name, keyword, floating subheading word, candidate term word] (4322)
33 "substance use".mp. [mp=title, abstract, heading word, drug trade name, original title, device manufacturer, drug manufacturer, device trade name, keyword, floating subheading word, candidate term word] (53340)
34 cannabis.mp. [mp=title, abstract, heading word, drug trade name, original title, device manufacturer, drug manufacturer, device trade name, keyword, floating subheading word, candidate term word] (56718)
35 marijuana.mp. [mp=title, abstract, heading word, drug trade name, original title, device manufacturer, drug manufacturer, device trade name, keyword, floating subheading word, candidate term word] (19644)
36 cocaine.mp. [mp=title, abstract, heading word, drug trade name, original title, device manufacturer, drug manufacturer, device trade name, keyword, floating subheading word, candidate term word] (72391)
37 cigarette*.mp. [mp=title, abstract, heading word, drug trade name, original title, device manufacturer, drug manufacturer, device trade name, keyword, floating subheading word, candidate term word] (130261)
38 smok*.mp. [mp=title, abstract, heading word, drug trade name, original title, device manufacturer, drug manufacturer, device trade name, keyword, floating subheading word, candidate term word] (575144)
39 alcohol.mp. [mp=title, abstract, heading word, drug trade name, original title, device manufacturer, drug manufacturer, device trade name, keyword, floating subheading word, candidate term word] (636429)
40 "drug use".mp. [mp=title, abstract, heading word, drug trade name, original title, device manufacturer, drug manufacturer, device trade name, keyword, floating subheading word, candidate term word] (174494)
41 meth*.mp. [mp=title, abstract, heading word, drug trade name, original title, device manufacturer, drug manufacturer, device trade name, keyword, floating subheading word, candidate term word] (13307047)
42 methamphetamine.mp. [mp=title, abstract, heading word, drug trade name, original title, device manufacturer, drug manufacturer, device trade name, keyword, floating subheading word, candidate term word] (23404)
43 Pharmaceutical misuse.mp. [mp=title, abstract, heading word, drug trade name, original title, device manufacturer, drug manufacturer, device trade name, keyword, floating subheading word, candidate term word] (7)
44 24 or 25 or 26 or 27 or 28 or 29 or 30 or 31 or 32 or 33 or 34 or 35 or 36 or 37 or 38 or 39 or 40 or 41 or 42 or 43 (14129847)
45 intervention.mp. [mp=title, abstract, heading word, drug trade name, original title, device manufacturer, drug manufacturer, device trade name, keyword, floating subheading word, candidate term word] (1054033)
46 prevent*.mp. [mp=title, abstract, heading word, drug trade name, original title, device manufacturer, drug manufacturer, device trade name, keyword, floating subheading word, candidate term word] (3066652)

47 preventive health services.mp. [mp=title, abstract, heading word, drug trade name, original title, device manufacturer, drug manufacturer, device trade name, keyword, floating subheading word, candidate term word] (1221)
48 prevention control.fs. (0)
49 46 or 47 or 48 (3066652)
50 18 and 23 and 44 and 45 and 49 (794)
51 art therap*.mp. [mp=title, abstract, heading word, drug trade name, original title, device manufacturer, drug manufacturer, device trade name, keyword, floating subheading word, candidate term word] (4902)
52 1 or 2 or 3 or 4 or 5 or 6 or 7 or 8 or 9 or 10 or 11 or 12 or 13 or 14 or 15 or 16 or 17 or 51 (955359)
53 23 and 44 and 45 and 49 and 52 (807)

****

APPENDIX 5: Searching Strategies of the Database Web of Science

Searching Strategies[1]	Number of Results
(Art OR Artist* OR Artwork* OR Art therap* OR Art* adj1 work* OR Photovoice* OR Photography OR Time* adj2 OR media OR Paint* OR Sculpture* OR Film* OR Movie* OR Video* OR Danc* OR Drama* OR Music* OR Theatre* OR Theater*) AND (adolescent OR Teen* OR Youth* OR Adolescen*) AND (Substance-related OR disorders OR Chemical dependence* OR Drug abuse OR Drug addiction OR Drug dependence OR Drug use disorder* OR Substance abuse* OR Pharmaceutical misuse OR Substance addict* OR Substance dependen* OR Substance use OR Cannabis OR Marijuana OR Cocaine OR Smok* OR Alcohol OR Drug use OR Meth* OR Methamphetamine) AND Intervention AND (Prevent* OR Preventive Health Services OR Prevention control)	2,480

[1] Please note that Web of Science now has terms limits (50) when conducting search.

APPENDIX 6: Searching Strategies of the Database APA PsycInfo
Database: APA PsycInfo <1806 to March Week 5 2021>
Search Strategy:
----
1 exp Art/ (13800)
2 (art* adj1 work*).mp. [mp=title, abstract, heading word, table of contents, key concepts, original title, tests & measures, mesh] (1527)
3 artist*.mp. [mp=title, abstract, heading word, table of contents, key concepts, original title, tests & measures, mesh] (16899)
4 artwork*.mp. [mp=title, abstract, heading word, table of contents, key concepts, original title, tests & measures, mesh] (2084)
5 photovoice*.mp. [mp=title, abstract, heading word, table of contents, key concepts, original title, tests & measures, mesh] (872)
6 photography.mp. [mp=title, abstract, heading word, table of contents, key concepts, original title, tests & measures, mesh] (3411)
7 (time* adj2 media).mp. [mp=title, abstract, heading word, table of contents, key concepts, original title, tests & measures, mesh] (335)
8 paint*.mp. [mp=title, abstract, heading word, table of contents, key concepts, original title, tests & measures, mesh] (8768)
9 sculpture*.mp. [mp=title, abstract, heading word, table of contents, key concepts, original title, tests & measures, mesh] (876)
10 film*.mp. [mp=title, abstract, heading word, table of contents, key concepts, original title, tests & measures, mesh] (20614)
11 movie*.mp. [mp=title, abstract, heading word, table of contents, key concepts, original title, tests & measures, mesh] (7553)
12 video*.mp. [mp=title, abstract, heading word, table of contents, key concepts, original title, tests & measures, mesh] (71287)
13 danc*.mp. [mp=title, abstract, heading word, table of contents, key concepts, original title, tests & measures, mesh] (9113)
14 drama*.mp. [mp=title, abstract, heading word, table of contents, key concepts, original title, tests & measures, mesh] (37442)
15 music*.mp. [mp=title, abstract, heading word, table of contents, key concepts, original title, tests & measures, mesh] (42126)
16 theatre*.mp. [mp=title, abstract, heading word, table of contents, key concepts, original title, tests & measures, mesh] (3022)
17 theater*.mp. [mp=title, abstract, heading word, table of contents, key concepts, original title, tests & measures, mesh] (2718)
18 1 or 2 or 3 or 4 or 5 or 6 or 7 or 8 or 9 or 10 or 11 or 12 or 13 or 14 or 15 or 16 or 17 (207553)
19 exp Adolescent/ (0)
20 adolescen*.mp. [mp=title, abstract, heading word, table of contents, key concepts, original title, tests & measures, mesh] (480984)
21 teen*.mp. [mp=title, abstract, heading word, table of contents, key concepts, original title, tests & measures, mesh] (24219)
22 youth*.mp. [mp=title, abstract, heading word, table of contents, key concepts, original title, tests & measures, mesh] (113316)

23 19 or 20 or 21 or 22 (537745)
24 exp Substance-Related Disorders/ (0)
25 chemical dependence.mp. [mp=title, abstract, heading word, table of contents, key concepts, original title, tests & measures, mesh] (337)
26 drug abuse.mp. [mp=title, abstract, heading word, table of contents, key concepts, original title, tests & measures, mesh] (56015)
27 drug addiction.mp. [mp=title, abstract, heading word, table of contents, key concepts, original title, tests & measures, mesh] (15320)
28 drug dependence.mp. [mp=title, abstract, heading word, table of contents, key concepts, original title, tests & measures, mesh] (3729)
29 "drug use disorder*".mp. [mp=title, abstract, heading word, table of contents, key concepts, original title, tests & measures, mesh] (1523)
30 substance abuse*.mp. [mp=title, abstract, heading word, table of contents, key concepts, original title, tests & measures, mesh] (44739)
31 substance addict*.mp. [mp=title, abstract, heading word, table of contents, key concepts, original title, tests & measures, mesh] (668)
32 substance dependen*.mp. [mp=title, abstract, heading word, table of contents, key concepts, original title, tests & measures, mesh] (3532)
33 "substance use".mp. [mp=title, abstract, heading word, table of contents, key concepts, original title, tests & measures, mesh] (65317)
34 cannabis.mp. [mp=title, abstract, heading word, table of contents, key concepts, original title, tests & measures, mesh] (13078)
35 marijuana.mp. [mp=title, abstract, heading word, table of contents, key concepts, original title, tests & measures, mesh] (12277)
36 cocaine.mp. [mp=title, abstract, heading word, table of contents, key concepts, original title, tests & measures, mesh] (22771)
37 cigarette*.mp. [mp=title, abstract, heading word, table of contents, key concepts, original title, tests & measures, mesh] (22468)
38 smok*.mp. [mp=title, abstract, heading word, table of contents, key concepts, original title, tests & measures, mesh] (64451)
39 alcohol.mp. [mp=title, abstract, heading word, table of contents, key concepts, original title, tests & measures, mesh] (126994)
40 "drug use".mp. [mp=title, abstract, heading word, table of contents, key concepts, original title, tests & measures, mesh] (35468)
41 meth*.mp. [mp=title, abstract, heading word, table of contents, key concepts, original title, tests & measures, mesh] (1087584)
42 methamphetamine.mp. [mp=title, abstract, heading word, table of contents, key concepts, original title, tests & measures, mesh] (6355)
43 Pharmaceutical misuse.mp. [mp=title, abstract, heading word, table of contents, key concepts, original title, tests & measures, mesh] (6)
44 24 or 25 or 26 or 27 or 28 or 29 or 30 or 31 or 32 or 33 or 34 or 35 or 36 or 37 or 38 or 39 or 40 or 41 or 42 or 43 (1277881)
45 intervention.mp. [mp=title, abstract, heading word, table of contents, key concepts, original title, tests & measures, mesh] (288630)
46 prevent*.mp. [mp=title, abstract, heading word, table of contents, key concepts, original title, tests & measures, mesh] (246911)

47 preventive health services.mp. [mp=title, abstract, heading word, table of contents, key concepts, original title, tests & measures, mesh] (4108)
48 [prevention control.fs.] (0)
49 46 or 47 or 48 (246911)
50 18 and 23 and 44 and 45 and 49 (350)
51 art therap*.mp. [mp=title, abstract, heading word, table of contents, key concepts, original title, tests & measures, mesh] (6188)
52 1 or 2 or 3 or 4 or 5 or 6 or 7 or 8 or 9 or 10 or 11 or 12 or 13 or 14 or 15 or 16 or 17 or 51 (210948)
53 23 and 44 and 45 and 49 and 52 (356)

****

APPENDIX 7: Searching Strategies of Grey Literature

No.	Websites, organizations and literature found by Google search	Number of relevant results
1	Canadian Institutes of Health Research	0
2	Canadian Institute for Health Information (CIHI)	0
3	Public safety Canada	0
4	Literature: School-Based Drug Abuse Prevention: Promising and Successful Programs	N/A
5	Youth.gov	0
6	Canadian Centre on Substance Abuse	0
7	Government of Canada	0
8	Public Health Ontario	0
9	Pan-Canadian Joint Consortium for School Health	0
10	Addiction Centre	0
11	Alberta Health Services	0
12	The Canadian Agency for Drugs and Technologies in Health (CADTH)	0
Grey Matters, published by CADTH, for more information, please visit https://www.cadth.ca/grey-matters-practical-tool-searching-health-related-grey-literature-0		
1	Health Technology Assessment (HTA) Agencies[1]: Health Organizations and Institutes Globally	0

[1] Selecting this category is because it contained the biggest number of health organizations and institutes globally, and this category is more relevant to the topic, compare with other categories.
